# Differential contribution of education through KIR2DL1, KIR2DL3, and KIR3DL1 to antibody‐dependent (AD) NK cell activation and ADCC

**DOI:** 10.1002/JLB.4A0617-242RRR

**Published:** 2019-01-30

**Authors:** Irene Lisovsky, Sanket Kant, Alexandra Tremblay‐McLean, Gamze Isitman, Zahra Kiani, Franck P. Dupuy, Louise Gilbert, Julie Bruneau, Naglaa H. Shoukry, Bertrand Lebouché, Nicole F. Bernard

**Affiliations:** ^1^ Research Institute of the McGill University Health Center (RI‐MUHC) Montreal Quebec Canada; ^2^ Division of Experimental Medicine McGill University Montreal Quebec Canada; ^3^ Centre de Recherche du Centre Hospitalier de l'Université de Montréal (CRCHUM) Montreal Quebec Canada; ^4^ Department of Family and Emergency Medicine Université de Montréal Montreal Quebec Canada; ^5^ Department of Family Medicine McGill University Montréal Québec Canada; ^6^ Chronic Viral Illness Service McGill University Health Centre Montreal Quebec Canada; ^7^ Division of Clinical Immunology McGill University Health Centre Montreal Quebec Canada

**Keywords:** ADCC, Ab‐dependent NK cell activation, KIR, NK cell education, NKG2A

## Abstract

The engagement of activating NK receptors (aNKR) stimulates NK cell activity, provided that interactions between inhibitory NK receptors (iNKR) with their HLA ligands do not override them. Abs bound to target cells can also activate NK cells by engaging the CD16 aNKR. NK cell education status is an important factor for Ab‐dependent NK cell activation (ADNKA) of some NK cell subsets. However, whether NK cell education also influences Ab‐dependent cellular cytotoxicity (ADCC) levels is not fully known. ADCC‐GranToxiLux (GTL) assays measured ADCC activity as the frequency of granzyme B positive (%GzB^+^) target cells. Target cells were anti‐HIV Immunoglobulin G (HIVIG)‐opsonized CEM‐NKr.CCR5 (CEM) cells. Lymphocytes and sorted single positive (SP) NKG2A^+^, KIR2DL1^+^, KIR2DL3^+^, and KIR3DL1^+^ NK cells, to self‐ and nonself HLA, were used as effectors in ADCC‐GTL assays to examine how education status influenced ADCC activity. ADNKA activity was assessed by stimulating lymphocytes with HIVIG‐opsonized CEMs and measuring the frequency of NK cell populations defined by their expression of iNKRs, along with IFN‐γ, CCL4, and CD107a functions. ADCC: the %GzB^+^ CEM cells generated by self‐ versus nonself HLA‐specific SPiNKR did not differ. ADNKA: More NK cells educated through KIR2DL1 and KIR3DL1, but not KIR2DL3, responded to ADNKA than their uneducated counterparts. CD16 engagement induced ADCC and ADNKA activity. With the proviso that groups’ sizes were small, our results support the notion that NK cell education does not influence ADCC levels but does contribute to ADNKA activity.

## Graphical abstract

NK cell education through inhibitory Killer Immunoglobulin‐like Receptors activates antibody‐dependent NK cell functions but mediate antibody‐dependent cellular cytotocitiy no better than their uneducated counterparts.

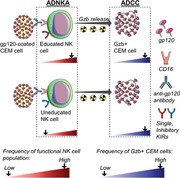

**Abbreviations**
%Fxn: percent of all functional cells
%GzB^+^: frequency of GzB positive
*h/*y: Homozygous *KIR3DL1* genotype encoding at least 1 high expression variant
*h/*y+B*57: Combined genotypes including genes encoding high expression KIR3DL1 and HLA‐B*57
ADCC: antibody‐dependent cellular cytotoxicity
ADCC‐GTL: antibody‐dependent cellular cytotoxicity GranToxiLux
ADNKA: Ab‐dependent NK activation
aNKRs: activating NK cell receptors
BnAb: broadly neutralizing antibodies
C1: HLA‐C group 1
C2: HLA‐C group 2
CEM: HIV Envelope gp120‐coated CEM‐NKr.CCR5 cells
Env: Envelope
FcR: Fc receptor
FCS‐A: forward scatter area
GTL: GranToxiLux
GWAS: Genome‐wide association studies
GzB: granzyme B
HIVIG: anti‐HIV Immunoglobulin G
Hmz: homozygotes
iKIR: Inhibitory Killer Immunoglobulin‐like receptor
iNKRs: inhibitory NK receptors
IQR: interquartile range
KIR: Killer Immunoglobulin‐like Receptor
KIR2DL1: 2DL1
KIR2DL2: 2DL2
KIR2DL3: 2DL3
KIR3DL1: 3DL1
MHC‐I: major histocompatibility complex class I
Rgp120: recombinant gp120
RT: room temperature
SP: single positive
SSC‐A: side scatter area

## 1 INTRODUCTION

Although there is great interest in developing an effective vaccine against HIV infection, the type of immune response that would be needed to confer protection in this context remains unclear. Although broadly neutralizing antibodies (BnAb) may be able to protect against infection, there are still significant challenges to inducing such antibodies (Abs) through vaccination.^1^, ^2^, ^3^ The capacity for BnAbs to protect humanized mice or rhesus macaques against challenge with HIV or simian/human immunodeficiency virus partially depends on their Fc region, which interacts with Fc receptors (FcR) on innate immune cells.^4^, ^5^, ^6^, ^7^, ^8^ One of these FcRs, FcRγIIIa (CD16), is found on NK cells, macrophages and monocyte subsets.^6^, ^9^, ^10^ CD16 engagement leads to NK cell activation, as measured by secretion of chemokines, cytokines and the release of cytotoxic granules that can initiate the lysis of target cells recognized by Abs bridging effector and target cells.^11^, ^12^ The activation of NK cells by Ab‐dependent stimuli and Ab‐coated target cell lysis by NK cells (Ab bridging NK and target cells) are often measured separately, but are frequently referred to in the literature as Ab‐dependent cellular cytotoxicity (ADCC). However, the former should be called Ab‐dependent NK cell activation (ADNKA), whereas the latter is more correctly referred to as ADCC.

In a post hoc analysis of the results of the RV144 Thai HIV vaccine trial, which conferred modest protection against HIV infection, ADCC activity was shown to be a correlate of protection.^13^, ^14^ Follow‐up analyses using systems serology that measured and correlated several HIV envelope (Env) specific Ab Fc‐dependent functions and features revealed that NK cell activation by Ab‐dependent stimulation that supported immune responses other than cellular cytotoxicity, were also correlated with protective responses.^15^, ^16^ Although this work underlines the value of Ab‐mediated NK cell activation as a correlate of protection, the influence of NK cell education on the ADNKA and ADCC activities of these NK cells is not fully characterized. Here, we used a standardized source of target cells, that is, HIV Env gp120 coated CEM.NKr.CCR5 and polyclonal anti‐Env Abs, to investigate the influence of NK cell education on ADNKA and ADCC.

Tolerance to self and the state of activation of NK cells is determined by an ontogenic process known as education, which requires the interaction of inhibitory NK receptors (iNKRs) with their cognate HLA ligands on neighboring cells.^17^, ^18^ Education is a complex process whereby functionality is tuned by the number of iNKRs engaged, the strength of interactions between iNKRs and their ligands and whether activating NK cell receptors (aNKRs) are also engaged.^19^, ^20^, ^21^, ^22^, ^23^, ^24^ NK cells lacking iNKRs for self‐HLA ligands remain uneducated and hyporesponsive.^25^ iNKRs include NKG2A and killer immunoglobulin‐like receptor (KIR)3DL1 (hereafter 3DL1), KIR2DL1 (2DL1), KIR2DL2 (2DL2), and KIR2DL3 (2DL3). NKG2A, a C‐type lectin receptor, forms a heterodimer with CD94 and interacts with nonclassical MHC class I (MHC‐I) HLA‐E molecules that present leader peptides from many MHC‐I proteins.^26^, ^27^ NKG2A binds peptides from the signal sequence of HLA‐A, ‐B, ‐C, and ‐G allotypes.^27^, ^28^ Recently, Horowitz et al. identified a dimorphism at position ‐21 of the HLA signal sequence that influences whether a nonamer peptide that includes this amino acid will preferentially bind HLA‐E to form a ligand for NKG2A/CD94 or will provide ligands for iKIR.^29^ The 3DL1 receptor interacts with a subset of HLA‐A and ‐B antigens that belong to the Bw4 group.^30^ Bw4 antigens differ from the remaining Bw6 variants at amino acids 77–83 of the HLA heavy chain.^31^ Bw6 isoforms do not interact with 3DL1 receptors such that 3DL1^+^ NK cells from individuals carrying no *Bw4* alleles are not educated through this receptor. The 2DL3 receptor, which is encoded at the same locus as 2DL2, interacts with HLA‐C group 1 (C1) variants that have an asparagine at position 80.^32^, ^33^, ^34^ The remaining HLA‐C variants, which belong to the C2 group, have a lysine at this position and are ligands for 2DL1 receptors on NK cells.^32^ The 2DL3 receptor can also bind certain allelic variants of C2, though with lower affinity than either 2DL1 or 2DL2.^33^, ^35^ Therefore, 2DL3^+^ NK cells from individuals expressing the C1 ligand are educated but remain uneducated or modestly educated through this receptor in individuals who do not carry a C1 group ligand. In contrast, 2DL1^+^ NK cells require the expression of the C2 ligand on neighboring cells to be educated through this iKIR.

Genome‐wide association studies (GWAS) found that genes influencing HIV viral load set point map to the *MHC‐I* region on chromosome 6, which encodes MHC‐I proteins that are also recognized by iKIR on NK cells.^36^ Both epidemiological and functional studies have implicated iKIRs, particularly 3DL1, in combination with certain HLA‐B variants in protection from HIV infection and slow disease progression in those already infected.^37^, ^38^ For example, when compared to *Bw6* homozygotes (hmz), co‐carriage of the homozygous *KIR3DL1* genotype encoding at least 1 high expression variant designated as *h/*y with HLA‐B*57 (*h/*y+B*57) is associated with the strongest effects on slow time to AIDS and HIV viral load control.^37^ NK cells from *h/*y+B*57 carriers, compared to those from *Bw6* hmz, have a superior functional potential upon stimulation with HLA null cells and ability to inhibit HIV replication through mechanisms that involve secretion of CC‐chemokines.^22^, ^39^, ^40^ An upstream region of HLA‐C that plays a role in determining HLA‐C expression levels was also associated with HIV control in individuals of European American origin in GWAS studies.^36^ While the mechanisms underlying this association are related to HLA‐C expression levels and the potency of CD8^+^ T cell recognition of HLA‐C‐HIV peptide complexes, the potential involvement of NK cells has not been excluded.^41^

Our group previously showed that NK cells from carriers of the educating *3DL1‐Bw4*, compared to those from carriers of noneducating *3DL1‐Bw6*, *KIR‐HLA* combinations generated similar levels of ADCC activity in target cells.^42^ This was despite a higher frequency of NK cells from *3DL1‐Bw4* carriers responding to ADNKA by secreting IFN‐γ and CCL4 and expressing CD107a than do NK cell from *3DL1‐Bw6* carriers.^42^ Here, we showed that PBMCs containing NK cells from carriers of the educating *KIR‐HLA* pairs *2DL1‐C2* and *2DL3‐C1*, compared to their noneducating counterparts, also generated similar frequencies of ADCC target cells. Purified NK cells, single positive (SP) for 2DL1, 2DL3, 3DL1, and NKG2A were used as effector cells in ADCC‐GranToxiLux (ADCC‐GTL) assays. The education status of SPiKIR^+^ cells had no impact of the frequencies of granzyme B positive (%GzB^+^) target cells. Using flow cytometry and a Boolean gating strategy, which identified NK cell populations expressing only one of the 2DL1, 2DL2/S2, 2DL3, 3DL1, or NKG2A receptors we showed that education through 3DL1 and 2DL1, but not 2DL3, conferred superior responsiveness to SPiNKR expressing cells in ADNKA assays compared to their noneducated counterparts.

## 2 MATERIALS AND METHODS

### 2.1 Ethics statement

This study was conducted in accordance with the principles expressed in the Declaration of Helsinki. It was approved by the Institutional Review Boards of the Comité d’Éthique de la Recherche du Centre Hospitalier de l'Université de Montréal and the Research Ethics Committee of the McGill University Health Centre. All individuals provided written informed consent for the collection of samples and subsequent analyses.

### 2.2 Study population and genotyping

Fifty‐four healthy HIV‐1–uninfected subjects were included in this study. Table S1 shows that the 47 donors previously tested in the ADCC‐GTL assay included 17 *C1/C1* hmz, 22 *C1/C2* heterozygotes and 8 *C2/C2* hmz.^42^ All were *KIR2DL1* carriers; of these, 30 (63%) co‐carried a *C2* group allele. Of the 45 who were *KIR2DL3* carriers, 37 (82.2%) co‐carried a *C1* group allele. These combined genotypes supported education through 2DL1 and 2DL3, respectively.

MHC‐I alleles were typed using commercial reagents (Atria Genetics, Inc., South San Francisco, CA, USA). Genotyping and allotyping of *3DL1* was performed as previously described.^38^, ^43^ Presence of *2DL1*, *2DL2/2DL3* and *2DS2* loci and *2DL2* and *2DL3* group alleles was determined by KIR region typing (One Lambda, Canoga Park, CA) and verified by PCR using specific primers and conditions described by Kulkarni et al.^44^

### 2.3 Cells and reagents

PBMCs were isolated from blood draws into vacutainers containing EDTA anti‐coagulant or from leukophoresis samples by density gradient centrifugation, as previously described.^40^, ^45^ Cells were frozen in 90% fetal bovine serum (FBS, Wisent, Inc., St Bruno, Quebec, Canada); 10% dimethyl sulfoxide (Sigma‐Aldrich, St. Louis, MO, USA) and stored in liquid nitrogen until use. Thawed PBMCs were rested overnight in RPMI 1640 medium supplemented with 10% FBS; 2 mM L‐glutamine; 50 IU/ml penicillin; and 50 mg/ml streptomycin (R10, all from Wisent). CEM.NKr.CCR5 (CEM; from the NIH AIDS Reagent Program, Division of AIDS, NIAID, NIH, Germantown MD, USA, from Dr. Alexandra Trkola) cells, recombinant gp120 (HIV‐1 Env rgp120 from HIV‐1_Bal_), and HIVIG pooled plasma from HIV infected donors (Catalog #3957, HIV‐IG from NABI and NHLBI) were obtained from the NIH AIDS Reagent Program.

### 2.4 ADCC‐GTL assay

PBMCs from 47 subjects were used as effector cells in ADCC‐GTL assays. The HLA‐A, ‐B, and ‐C allotypes of the 47 subjects has been previously reported.^42^ The ADCC‐GTL assays were performed as previously described.^42^, ^46^ Briefly, rgp120 coated CEM target cells were labeled with TFL4 and the viability marker NFL1 (both from OncoImmunin, Inc., Gaithersburg, MD, USA). Target cells (T) were plated at 10^4^ cells/well in 96‐well V‐bottomed plates with effector cells at an E:T of 30:1 with the GzB substrate supplied by the manufacturer. Following incubation for 5 min at room temperature (RT), HIVIG was added (or not, as a control) at a concentration of 50 μg/ml (a 1:1000 dilution). The samples were then pelleted by centrifugation and incubated for 1 h at 37°C in a humidified 5% CO_2_ incubator. Cells were washed, resuspended in wash buffer (supplied by OncoImmunin, Inc., as part of the GTL kit) and acquired with a calibrated LSRFortessa flow cytometer using a high throughput system (BD Biosciences, San Jose, CA, USA).

Cells from 8 subjects were also used as effector cells in a modified ADCC‐GTL assay. Table 1 provides information on the *KIR* genes and *HLA* allotypes carried by these individuals. NK cells were isolated from 1 × 10^9^ PBMCs using an immunomagnetic negative selection human NK cell enrichment kit (STEMCELL Technologies, Vancouver, British Columbia, Canada). Following isolation, cells were 93 ± 7.2% CD56^+^. These NK cells were stained for viability using the UV Live/Dead fixable dead cell stain kit (Invitrogen Burlington, ON, Canada) and for surface markers using anti‐CD3‐BV785 (OKT3, BioLegend, San Diego, CA), anti‐CD56‐PE‐Cy7 (REA196), anti‐KIR2DL1‐APC‐Cy7 (REA286), anti‐KIR2DL3‐PE (REA147), anti‐KIR3DL1‐VioBlue (REA1005), and anti‐NKG2A‐FITC (REA110) (all from Miltenyi Biotec, Auburn, CA, USA) for 20 min at 4°C. After staining cells were washed and resuspended in PBS; 2% HEPES (Wisent) buffer; and sorted using a FACSAria instrument (BD Biosciences). Live, singlet, CD3^−^CD56^Dim^ cells were gated on and from these, 4 populations were recovered: NKG2A^+^2DL1^−^2DL3^−^3DL1^−^ (SPNKG2A^+^), 2DL1^+^2DL3^−^3DL1^−^NKG2A^−^ (SP2DL1^+^), 2DL3^+^2DL1^−^3DL1^−^NKG2A^−^ (SP2DL3^+^), and 3DL1^+^2DL1^−^2DL3^−^NKG2A^−^ (SP3DL1^+^). These populations were washed, counted and used as effector cells in ADCC‐GTL assays as described in the previous section at E:Ts ranging from 10:1 to 1:1. PBMC from these subjects, cells were stained using the same cell surface specific Ab panel as described above, fixed with 2% paraformaldehyde, permeabilized with 0.125% saponin and stained intracellularly with anti‐GzB‐BV510 (GB11) and anti‐granule associated perforin‐AlexaFluor‐647 (δG9, both from BD Biosciences).^47^ An BV510 conjugated isotype control (BD Biosciences) and fluorescence minus one (FMO) staining were used as negative controls for GzB and perforin staining, respectively. The same gating strategy was used to examine the 4 populations of SPiNKR^+^ NK cells for the frequency and MFI of GzB^+^ and perforin^+^ cells. In a separate experiment, cell surface staining was performed using this Ab cocktail with the addition of anti‐CD16 (REA368, Miltenyi Biotec) in order to obtain information on the frequency of NK cell populations expressing SPiNKR and CD16.

**Table 1:** Study population HLA allotypes and presence of KIR2DL1, KIR2DL2, KIR2DS2, KIR2DL3, and KIR3DL1 genes

	Bw4/62	C1/C2	HLA‐A		HLA‐B		HLA‐C		2DL1	2DL2	2DS2	2DL3	3DL1
**2** a	Bw4^b^	C1/C2^c^	02:01	24:02	44:02	51:01	05	08	+^d^	+	+	+	+
**3**	Bw4/6	C1/C2	02:01	31:01	27:01	40:01	03:02	15:02	+	‐	‐	+	+
**5** e	Bw4/6	C1/C2	02:01	02:01	08:01	44:02	01:01	04:01	+	‐	‐	+	+
**6** f	Bw4	C1/C2	31:01	68:01	27:08	51:01	02:02	12:03	+	‐	‐	*	+
**12** e	Bw4/6	C1/C1	01:01	23:01	14:01	38:05	08:02	12:03	+	‐	‐	+	+
**21** f	Bw4/6	C2/C2	02:01	02:01	07:02	57:01	05	06:02	+	‐	‐	+	+
**23**	Bw6	C1/C1	02:01	02:01	18:01	40:01	03:04	07:01	+	‐	‐	+	+
**29**	Bw6	C1/C1	02:01	02:01	07:02	08:01	07	07	+	+	+	+	+
**30** f	Bw6	C1/C2	02:01	30:02	07:02	35:01	04:01	07:02	+	‐	‐	+	+
**31**	Bw6	C1/C1	02:01	03:01	07:02	40:01	03:02	07:10	+	+	+	+	+
**32** f	Bw6	C1/C2	03:01	11:01	07:02	35:01	04:01	07:02	+	‐	‐	+	+
**38**	Bw6	C1/C2	02:01	33:03	15:01	35:08	03:03	04:01	+	+	+	+	+
**34**	Bw6	C1/C1	01:01	02:01	08:01	40:01	03:02	07:01	+	+	+	+	+
**44**	Bw4/6	C2/C2	01:01	02:01	15:01	57:01	05	06:02	+	+	‐	+	+
**45**	Bw4	C1/C2	01:01	02:01	38:01	57:01	06:02	12:03	+	‐	‐	+	+
**48**	Bw4/6	C1/C2	01:01	03:01	14:02	57:01	06:02	08:02	+	+	+	+	+
**49**	Bw4	C1/C2	01:01	26:01	38:01	57:01	06:02	12:03	+	‐	‐	+	+
**50**	Bw4	C2/C2	02:02	30:02	53:01	57:03	04:01	18	+	+	+	+	+
**51**	Bw4/6	C2/C2	01:01	68:02	18:01	57:01	05:01	06:02	+	+	+	+	+
**52** e	Bw6	C2/C2	02:01	02:01	35	40	04:01	04:01	+	‐	‐	+	‐
**53** e	Bw6	C1/C1	03:01	03:01	18:01	35	03:03	16:01	+	‐	‐	+	‐
**54** e	Bw4/6	C1/C1	02:01	24:02	39:01	51:01	07:02	07:02	+	‐	‐	+	‐

a Subject codes from #2 through #50 are the same as those used in Table S1.

b Bw4 = Bw4 homozygote, Bw6 = Bw6 homozygote with no Bw4 at the HLA‐A locus, Bw4/6 = carriers of a Bw4 and Bw6 allele.

c C1/C1 = HLA‐C1 group homozygotes, C2/C2 = HLA‐C2 group homozygotes, C1/C2 = carriers of both a C1 and C2 group allotype.

d “‐” = absent; “+” = present.

e Subjects contributing PBMC used to isolate single positive inhibitory NK receptor positive (SPiNKR^+^) NK cells for use as effector cells for ADCC‐GranToxuLux (ADCC‐GTL) assays only.

f Subjects contributing PBMC used in both antibody‐dependent NK cell activation assays and to isolate SPiNKR^+^ for use as effector cells for ADCC‐GTL assays.

### 2.5 ADNKA assay

Seventeen subjects were included in studies analyzing the role of NK cell education in ADNKA. Table 1 shows for each of these subjects their *Bw4/Bw6* and *C1/C2* generic genotypes, their HLA‐A, ‐B and ‐C allotypes and which *KIR* genes whose gene products were stained for they carried. Stimulation of 2 × 10^6^ PBMCs with rgp120‐coated CEM cells were performed as previously described.^42^ Briefly, PBMCs and rgp120‐coated CEM cells prepared as above were cocultured at a 10:1 ratio in the presence or absence of 50 ug/ml (1:1000 dilution) of HIVIG and with anti‐CD107a‐PE‐CF594 (H4A3, BD Biosciences) for 6 h in a humidified 5% CO_2_ incubator. Brefeldin A (5 mg/ml; Sigma‐Aldrich) and monensin (6 mg/ml, Golgi Stop; BD Biosciences) were added 30 min into the coculture. Cells were stained for viability using the UV Live/Dead fixable dead cell stain kit (Invitrogen) and surface markers using anti‐CD3‐BV785 (OKT3), anti‐CD56‐BV711 (HCD56), anti‐2DL2/S2‐FITC (DX27), anti‐3DL1‐BV421 (DX9, all from BioLegend), anti‐2DL1‐AF700 (143211), anti‐2DL3‐PE (180701; both from R&D Systems, Minneapolis, MN), and anti‐NKG2A‐PECy7 (Z199, Beckman Coulter, Mississauga, ON, Canada). Cells were then washed, fixed and permeabilized according to the manufacturer's directions (Invitrogen), stained for intracellular IFN‐γ and CCL4 using anti‐IFN‐γ‐BV605 (B27, BD Biosciences) and anti‐CCL4‐APC (24006, R&D Systems).

### 2.6 Flow cytometry analysis

For ADNKA experiments, between 4 × 10^5^ and 1 × 10^6^ total events were acquired for each sample using a calibrated LSRFortessa flow cytometer (BD Biosciences). Single stained control beads (CompBead; BD Biosciences) were used in every experiment to calculate compensation.

Boolean gating was used to identify the frequency of NK cells having surface markers defining NK cell populations with each of the 7 possible functional profiles: tri‐functional (CD107a^+^IFN‐γ^+^CCL4^+^), bi‐functional (any combination of 2 functions) and mono‐functional. Total responsiveness was defined as the sum of the frequencies of tri‐, bi‐, and mono‐functional NK cells. Total CD107a, total IFN‐γ, and total CCL4 were defined as the sum of all functional subsets that included these functions. Analysis was performed using FlowJo software V9.8 (TreeStar, Ashland, OR, USA). The data obtained were corrected for background using PBMCs stimulated with rgp120‐coated CEM cells in the absence of HIVIG.

### 2.7 Statistical analysis

Analysis was performed using GraphPad Prism 6 software (GraphPad, La Jolla, CA, USA). Friedman tests with Dunn's post‐tests were used to analyze the significance of differences between the frequencies of functional subsets among the NK cell populations gated on inclusively and to assess the significance of differences between the %GzB^+^ CEM cells generated using SPiNKR or total NK cells as effector cells in ADCC‐GTL assays when more than 2 matched groups were compared. Wilcoxon matched pairs tests were used to assess the significance of comparisons for 2 matched data sets. Mann‐Whitney tests were used to assess the significance of comparisons for 2 unmatched data sets. Results are displayed as median and interquartile range (IQR). *P*‐values <0.05 were considered significant.

### 2.8 Online supplemental material

Table S1 provides information on the *HLA‐C C1/C2* group genotypes, presence of a *KIR2DL1* gene and *KIR2DL2/2DL3* group allotype and the presence of educating KIR2DL/HLA‐C pairs for the subjects included in this study. Table S2 provides information on the frequency of NK cells, NK cell subsets, results of ADCC‐GTL experiments using PBMCs from study subjects and whether cells from these individuals were used in ADNKA experiments. Figure S1A shows the gating strategy used to assess the %GzB^+^ CEM cells in ADCC‐GTL assays. Figure S1B shows the association between the %GzB^+^ CEM cells generated in ADCC‐GTL assays and the percentage loss of gp120 coated CEM cells in a 6 h ADCC assay using PBMCs as effector cells and HIVIG as a source of Ab. Figure S2 shows the gating strategy used to obtain SPiNKR by sorting and the staining of these SPiNKR cells for intracellular GzB and perforin. Figure S3 shows the %GzB^+^ cells generated using NK cell effector cells (E) and opsonized gp120 coated CEM cells as target (T) cells at E:T ranging from 10:1 to 1.25:1. Figure S4 shows the strategy used to gate on NK cell populations expressing defined iNKR for the purpose of assessing the frequency of their functional subsets.

## 3 RESULTS

### 3.1 The effect of NK cell education through 2DL1 and 2DL2/2DL3 on the ADCC‐GTL assay readout

**Figure 1:**
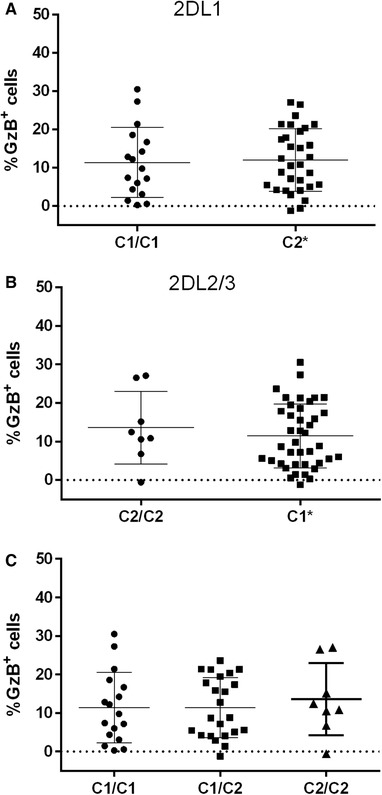
**The influence of NK cell education via KIR2DL1 (2DL1) and KIR2DL2/3 (2DL2/3) on the frequency of granzyme B positive (%GzB^+^) target cells generated in the ADCC‐GTL assay**. (A) The %GzB^+^ cells generated by PBMC from 2DL1^+^ individuals co‐expressing HLA‐C2 (i.e., educated) or not (i.e., uneducated HLA‐C1 homozygotes). (B) The %GzB^+^ cells generated by PBMC from 2DL2/3^+^ individuals co‐expressing HLA‐C1 (i.e., educated) or not (i.e., uneducated, HLA‐C2 homozygotes,). (C) The %GzB^+^ cells generated by PBMC from individuals expressing the 3 possible HLA‐C genotypes. C2* = both homozygotes and heterozygotes for C2; C1* = both homozygotes and heterozygotes for C1. The results presented in this figure are a re‐analysis of data previously published in reference 42

An ADCC‐GTL assay was used to measure %GzB^+^ CEM target cells generated by PBMC effector cells in the presence of HIVIG.^46^ Figure S1A shows the gating strategy used to determine the %GzB^+^ target cells. Figure S1B shows that the %GzB^+^ CEM cells generated in an ADCC‐GTL assay positively correlated with the percent loss of gp120 coated CEM target cells when PBMCs were used as effector cells and HIVIG as a source of anti‐gp120 specific Ab.^48^ Using the ADCC‐GTL assay, we previously tested a panel of PBMC effector cells from HIV uninfected subjects stratified according to whether they carried the *3DL1‐Bw4* or *3DL1‐Bw6* genotype combinations.^46^, ^49^ Figure 1 shows a reanalysis of these results stratified according to whether study subjects carried genotype combinations encoding other iKIR‐HLA combinations supporting education (*2DL1‐C2/C2 + 2DL1‐C1/C2, or 2DL2/2DL3‐C1/C2 + 2DL2/2DL3‐C1*) versus not (*2DL1‐C1/C1* or *2DL2/2DL3‐C2/C2*). No between‐group differences were detected for the %GzB^+^ cells generated by effector cells from carriers of *2DL1*, *2DL2*, or *2DL3* gene combinations that supported, versus not, NK cell education (Fig. 1A, B, *P* > 0.4, Mann‐Whitney tests). Furthermore, stratifying ADCC‐GTL results by the *C1/C1*, *C1/C2* and *C2/C2* genotypes of the effector cells showed no significant between‐group differences (Fig. 1C). Thus, we found no evidence that effector cells from subjects with educating iKIR‐HLA combinations were better than those from subjects without these combinations at supporting ADCC activity.

### 3.2 Populations of educated and uneducated SPiKIR NK effector cells generate similar %GzB^+^ CEM cells in the ADCC‐GTL assay

**Figure 2:**
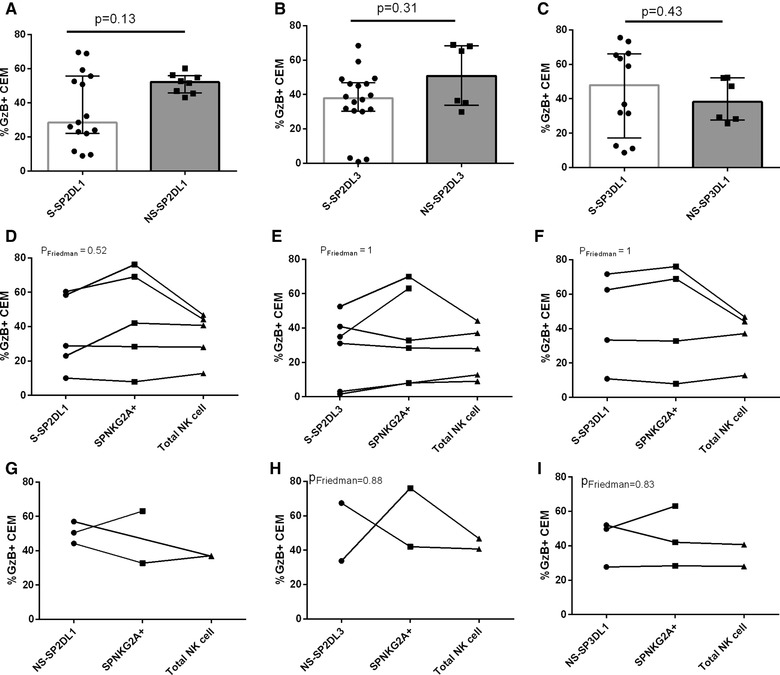
**The influence of NK cell education status of NK populations single positive for inhibitory NK receptors (SPiNKRs) on the frequency of granzyme B positive (%GzB^+^) target cells generated in ADCC‐GTL assays**. Panels A‐C show the %GzB^+^ CEM cells on the *y*‐axis generated in an ADCC‐GTL assay using SPiNKR to self‐HLA (i.e., educated) and nonself‐HLA (uneducated) as effector cells. SPiNKR effectors cells were KIR2DL1^+^ (2DL1) (A), KIR2DL3^+^ (2DL3) (B) and KIR3DL1^+^ (3DL1) (C). Bar heights and error bars represent the median and interquartile range %GzB^+^ CEMs for each data set. In panels D‐I, the *y*‐axis shows the %GzB^+^ CEM cells generated in an ADCC‐GTL assay using effector cells that were self‐SPiNKR^+^ (D‐F) or nonself‐SPiNKR (G‐I) compared to SPNKG2A^+^ cells and total NK cells from the same individual tested at the same effector to target cell ratios. SPiNKR effector cells were 2DL1^+^ (D, G), 2DL3^+^ (E, H) and 3DL1^+^ (F, I). Paired analyses (Friedman tests) compared within‐individual values for the %GzB^+^ CEM cells

The absence of an effect of NK cell education in the ADCC‐GTL assay when PBMCs are used as effector cells could have several explanations. For example, subtle differences may have been missed due to there being an insufficient number of study subjects to detect differences. Because PBMCs were used as effector cells for these experiments rather than isolated NK cells, it is possible the between‐subject differences in NK cell frequency could have influenced the conclusions drawn from these experiments. Despite stratifying ADCC results according to whether effector cells came from an individual expressing an iKIR to a self‐HLA ligand (self‐iKIR), the carriage of other combinations of self‐iKIR co‐expressed on the same NK cells or in the same pool of effector cells could contribute to ADCC‐GTL activity in a manner that could mask the effect of NK cell education through individual self‐iKIR on the %GzB^+^ CEM generated in this assay. To address this point, we isolated NK cells and sorted them into CD56^Dim^ populations expressing SPiNKR. Figure S2 depicts the gating strategy used to isolate SPiNKR cells from total NK cells. Table 2 provides information on the E:Ts used for these ADCC‐GTL assays for each donor and the education status of each donor's SPiKIRs. SPiNKR NK cells supported higher levels of %GzB^+^ CEM cells in the presence, compared to the absence, of HIVIG. This was the case whether effector cells originated from an individual educated, versus not, for each iKIR and for SPNKG2A and total NK cells (not shown). Figure 2A‐C shows the %GzB^+^ CEM cells generated by self‐ versus nonself‐SPiKIR. Differences in the %GzB^+^ CEM cells generated in ADCC‐GTL assays using self‐ and nonself‐SP2DL1^+^, SP2DL3^+^, and SP3DL1^+^ NK cells were not significant (Mann‐Whitney tests).

**Table 2:** Education status of single positive NK cell effector cells used in ADCC‐GTL assays and effector to target cell ratio used for each donor

Subject ID^a^	E:T^b^	KIR2DL1^c^	KIR2DL3^c^	KIR3DL1^c^
5	10:1	Yes	Yes	Yes
6	2:1	Yes	Yes	Yes
12	2.5:1	No	Yes	Yes
21	10:1	Yes	No	Yes
30	5:1	ND^d^	Yes	ND^d^
32	1:1	Yes	Yes	No
52	4:1	Yes	No	No
53	4:1	No	Yes	No
54	10:1	No	Yes	ND^e^

a Codes are the same as used in Tables 1 and S1.

b Effector to target cell ratio used.

c NK cell educated (Yes) or not educated (No) through this inhibitory NK receptor.

d Not done due to insufficient numbers of cells.

e Not done because subject 54 was a KIR3DS1 homozygotes with no KIR3DL1 receptors.

Due to the low frequency of SPiNKR populations we were unable to use the same E:T for all ADCC‐GTL experiments for all study subjects. Table S2 shows for 6 of the study subjects, the frequency of the SPiNKR populations present in total NK cells and the frequency of each of these SPiNKR cells that was CD16^+^. There was the possibility that the comparisons in ADCC‐GTL results generated by self‐ and nonself‐SPiKIR^+^ NK cells were influenced by differences in the distribution of E:Ts used in the self‐ and nonself‐SPiKIR groups. To control for this, we performed the ADCC‐GTL assay using total NK cells as effectors at E:T ranging from 10:1 to 1.25:1. Following background correction there were no differences observed in the %GzB^+^ CEM cells generated within this E:T range (Fig. S3). At lower E:Ts the %GzB^+^ CEM cell declined in a dose‐dependent manner. We found no correlation between the E:Ts used to test the effector activity of SPiKIR cells and the %GzB^+^ CEM generated (not shown).

SPiNKR and total NK cells isolated from individual study subjects were tested at the same E:Ts. As another control, we examined %GzB^+^ CEM generated by self‐SPiKIR, SPNKG2A, and total NK cells at these E:T. Figure 2D‐F show that SPiKIR generated similar %GzB^+^ CEM cells as matched SPNKG2A cells and that these values did not differ from that generated by total NK cells (*P* > 0.05 for all, Friedman tests). A comparison of the differences in the %GzB^+^ CEM cells generated using nonself‐SPiKIR^+^, versus SPNKG2A^+^ and total NK cells as effector cells (Fig 2G‐I) also revealed no significant between group differences in paired comparisons. Together, these results suggest that if NK cell education is playing a role in ADCC activity observed at the target cell level, the influence is modest and less important than the positive signaling arising from CD16 engagement.

**Figure 3:**
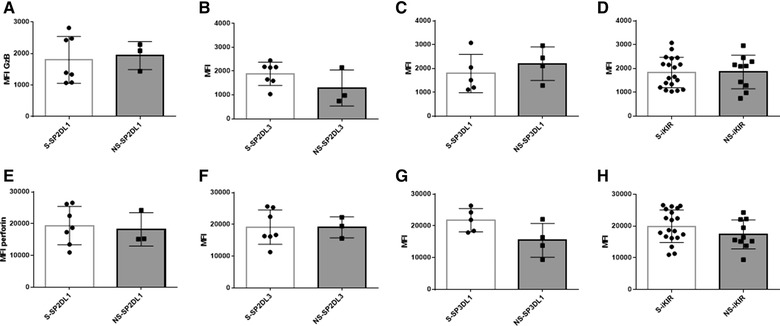
**The mean fluorescence intensity (MFI) of intracellular granzyme B (GzB) and perforin in NK populations single positive for inhibitory Killer Immunoglobulin‐like receptors (SPiKIRs) to self‐SPiKIR (educated) versus nonself‐iKIR (uneducated)**. The *y*‐axis shows the MFI of intracellular GzB (A‐D) and perforin (D‐F) in S‐SPiKIR^+^ and NS‐SPiKIR^+^ NK cell subset effector cells positive for KIR2DL1 (2DL1) (A, E), KIR2DL3 (2DL3) (B, F) and KIR3DL1 (3DL1) (C, G) and all 3 iKIR combined (D, H). Bar heights and error bars represent the median and interquartile range of GzB and perforin MFI. Mann‐Whitney tests were used to compare groups. No significant between group differences were found

To determine whether educated and uneducated NK cells differed in terms of the density of GzB and perforin in their intracellular granules, we negatively selected NK cells, stained them for cell surface markers, including iNKR and intracellularly for GzB and perforin. SPiKIR and SPNKG2A NK cells were then gated on to examine the mean fluorescence intensity (MFI) of their intracellular GzB and granule associated perforin (Fig S2). No significant differences were observed in the MFI (or percentage [not shown]) of GzB or perforin in educated versus uneducated SPiKIRs. This was the case whether NK cells expressing individual iKIRs or results for the 3 iKIRs combined were considered (Fig. 3). Levels of GzB and perforin in SPNKG2A^+^ NK cells did not differ significantly from those in SPiKIR^+^ NK cells (not shown).

### 3.3 Differential functional responses of NK cell populations to ADNKA

**Figure 4:**
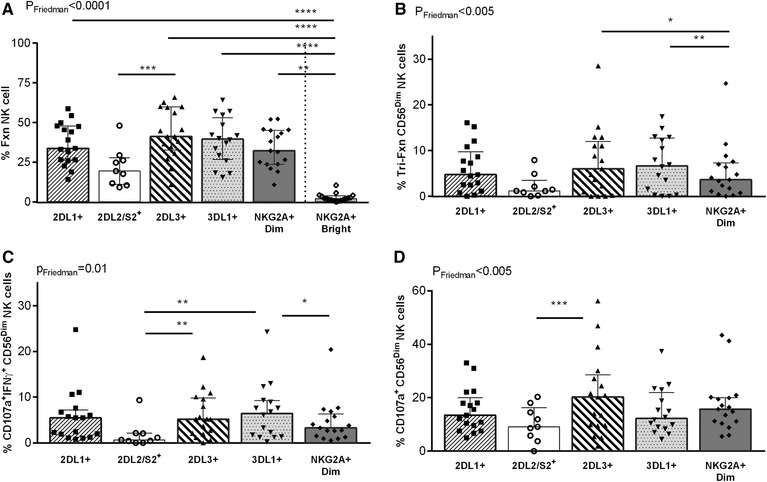
**Anti‐HIV antibody‐dependent NK cell activation (ADNKA) induced responses in CD56^Bright^NKG2A^+^ and CD56^Dim^ NK cell populations**. The frequency of ADNKA in NK cell populations characterized by total responsiveness (A), tri‐functional (B), CD107a^+^IFN‐γ^+^ (C) and CD107a^+^ (D) response profiles is shown on the *y*‐axis for CD56^Dim^2DL1, 2DL2/S2, 2DL3, 3DL1, NKG2A and CD56^Bright^NKG2A populations. The percent of all functional cells (%Fxn) was defined as the sum of the frequencies of all possible combinations of CD107a expression and IFN‐γ and CCL4 secretion. Given the low total responsiveness of the CD56^Bright^NKG2A population, it was excluded from further analysis. Each data point represents a single individual. Bar heights and error bars represent the median and interquartile range for each group. *P < 0.05, **P < 0.01, ***P < 0.001

We next analyzed responses to ADNKA of 6 different NK cell populations: CD56^Bright^ NKG2A^+^ and CD56^Dim^ 2DL1^+^, 2DL2/S2^+^, 2DL3^+^ 3DL1^+^ and NKG2A^+^ populations. For these experiments, we used the inclusive gating strategy shown in Fig. S4A to gate on these NK cell populations. In other words, CD56^Dim^ NK cell populations identified as NKG2A^+^ are not SP for this receptor and may also co‐express 2DL1, 2DL2/S2, 2DL3^+^, and/or 3DL1^+^. The functional responses of these NK cell populations were analyzed by Boolean gating. Figure S4B shows an example of the distribution of the functional subjects following stimulation with opsonized and unopsonized gp120 coated CEM cells. The frequency of cells responding to ADNKA was lower for CD56^Bright^NKG2A^+^ cells compared to each of the CD56^Dim^ NK populations, except for the CD56^Dim^2DL2/S2^+^ cells (Fig. 4A, *P* ≤ 0.009 for all comparisons, Dunn's post‐test). The frequency of CD56^Bright^NKG2A^+^ and CD56^Dim^2DL2/S2^+^ NK cells responding to ADNKA did not differ significantly (Fig. 4A, *P* > 0.9, Dunn's post‐test). Given that CD56^Bright^ NK cells, which are considered less mature than CD56^Dim^ NK cells, expressed low levels of iKIRs, a lower frequency of CD16^+^ (46.3 [31.8, 55.7]%) and that they exhibited low functionality in response to ADNKA, we excluded this population from further analyses of the functional subsets detected by Boolean gating.^49^, ^50^, ^51^

Significant differences in the frequency of responsive NK cell populations included fewer CD56^Dim^2DL2/S2^+^ than CD56^Dim^2DL3^+^ cells that were CD107a^+^IFN‐γ^+^ and mono‐functional for CD107a^+^ (Fig. 4C and D, respectively, (*P* ≤ 0.001 for both, Dunn's post‐tests), fewer CD56^Dim^2DL2/S2^+^ than 3DL1^+^ NK cells that were CD107a^+^IFN‐γ^+^ (Fig. 4C, *P* < 0.001, Dunn's post‐test), fewer tri‐functional CD56^Dim^NKG2A^+^ than 2DL3^+^ and 3DL1^+^ NK cells (Fig. 4B, *P* ≤ 0.01 for both, Dunn's post‐test) and fewer CD56^Dim^NKG2A^+^ than 3DL1^+^ cells that were CD107a^+^IFN‐γ^+^ (Fig. 4C, *P* = 0.01, Dunn's post‐test). Together, these results highlight the low responsiveness of the less differentiated CD56^Bright^NKG2A^+^ NK cell population to ADNKA. Of the more mature CD56^Dim^ NK cell populations a lower frequency of 2DL2/S2^+^ NK cells responded to ADNKA than the other CD56^Dim^ populations.

### 3.4 Impact of education through 3DL1 and 2DL1 on ADNKA

The differential functional responses induced by ADNKA prompted us to investigate the role of NK cell education on the frequency of cells responding to ADNKA. For these analyses, we examined CD56^Dim^ NK cells by applying an exclusive gating strategy to focus on cells positive for the iKIR 2DL1, 2DL3, or 3DL1 to the exclusion of the other NKRs stained for. The exclusive gating was achieved by applying a 5‐way Boolean analysis on the iNKR gates derived from the CD56^Dim^ population. We then analyzed the function of NK cell populations that expressed 3DL1, 2DL1, or 2DL3 to the exclusion of the other NKRs stained for (e.g., SP2DL1^+^ were 2DL1^+^2DL2/S2^−^2DL3^−^3DL1^−^NKG2A^−^). To assess the role of NK cell education in responses to ADNKA we compared the frequency of CD56^Dim^ NK cells expressing SP3DL1, SP2DL1 and SP2DL3 from individuals who co‐carried or not an HLA ligand for these iKIR.

**Figure 5:**
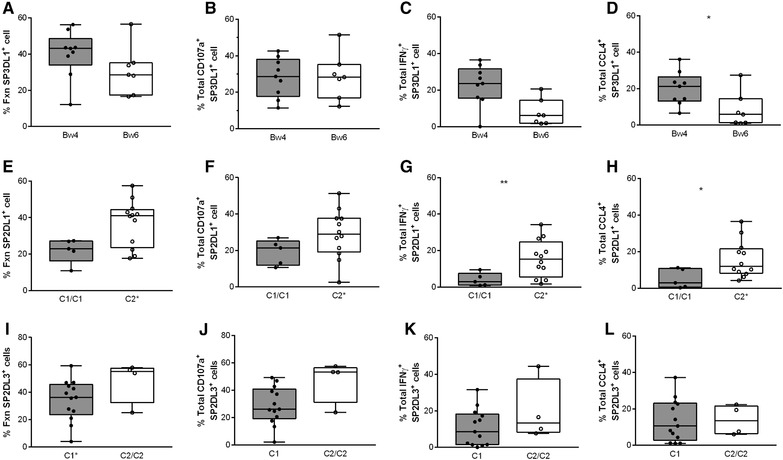
**Antibody‐dependent NK cell activation (ADNKA) of stimulated CD56^Dim^ single positive (SP) KIR3DS1^+^ (SP3DL1^+^), KIR2DL1^+^ (SP2DL1^+^) and KIR2DL3^+^ (SP2DL3^+^) NK cells from carriers of HLA allotypes able to educate, versus not, NK cells through these Inhibitory Killer Immunoglobulin‐like Receptors (iKIRs)**. The *y*‐axis shows the frequency of stimulated CD56^Dim^SP3DL1^+^ NK cells from carriers of a *Bw4* allele versus *Bw6* homozygotes (A‐D), the frequency of stimulated CD56^Dim^SP2DL1^+^ NK cells from carriers of a *C2* allele versus *C1* homozygotes (E‐H) and the frequency of stimulated CD56^Dim^SP2DL3^+^ NK cells from carriers of a *C1* allele versus *C2* homozygotes (I‐L) characterized by total responsiveness (A, E, I), total CD107a (B, F, J), total IFN‐γ (C, G, K), and total CCL4 (D, H, L) response profiles. Each data point represents a single individual. The upper and lower limits of the bars and error bars in these bar and whisker plots represents the interquartile range and maximum/minimum values whereas the line in the bars represents the median value for the group. The significance of comparisons of the frequency of ADNKA in NK cell populations educated, versus not, through these iKIRs is show by “*” = *P* ≤ 0.05 or “**” = *P* = 0.01). %Fxn = percent of all functional cells; SP = single positive cells expressed the indicated iKIR to the exclusion of all other inhibitory NK cell receptors measured

The frequency of SP3DL1^+^ NK cells characterized by the sum of all, total IFN‐γ and total CCL4 responses to ADNKA was greater when cells came from a subject carrying, versus not, a *Bw4* allele (Fig. 5A‐D). These differences achieved statistical significance for IFN‐γ and CCL4 secretion (Fig. 5C, D, respectively, *P* ≤ 0.03 for both comparisons, Mann‐Whitney tests). The frequency of SP2DL1^+^ NK cells characterized by the sum of all, total CD107a, total IFN‐γ, and total CCL4 responses to ADNKA was greater when from subjects who carried a *C2^+^* than a *C1/C1* hmz genotype (Fig. 5E‐H). These differences achieved statistical significance for total IFN‐γ and total CCL4 secretion (Fig. 5G, H, respectively, *P* ≤ 0.04 for both comparisons, Mann‐Whitney tests). Thus, NK cell education through 3DL1 and 2DL1 resulted in a higher frequency of NK cells able to secrete IFN‐γ and CCL4 in response to ADNKA than NK cells not educated through these iKIR.

### 3.5 Similar frequencies of educated versus uneducated SP2DL3^+^ NK cells respond to ADNKA

On the other hand, comparisons of the frequency of SP2DL3^+^ NK cells from C1 versus C2/C2 carriers characterized by the sum of total IFN‐γ and total CCL4 secretion responses revealed no significant difference (Fig. 5I, K, L, *P* ≥ 0.1 for all, Mann‐Whitney). Comparisons of the frequency of total CD107a^+^ SP2DL3^+^ NK cells from subjects carrying an educating versus noneducating 2*DL3‐HLA‐C* combination trended towards being significantly lower (Fig. 5J, *P* = 0.06, Mann‐Whitney). Thus, there was no evidence that education through 2DL3 increased the frequency of SP2DL3^+^ NK cells to ADNKA.

## 4 DISCUSSION

In this study, we found no differences in ADCC activity as measured by the %GzB^+^ targets generated in an ADCC‐GTL assay between conditions where effector cells included NK cells from subjects educated or not through 2DL1 or 2DL2/2DL3, as was previously reported for education through 3DL1.^42^ We showed that purified SPiKIR^+^ cells originating from carriers of HLA allotypes that did, versus did not, support the education of the SPNK populations behaved similarly in terms of their ability to generate GzB^+^ CEM cells. An examination of the functional profiles of six NK cell populations from HIV‐uninfected donors induced by anti‐HIV ADNKA revealed that CD56^Bright^NKG2A^+^ NK cells were the least responsiveness to this stimulus. The frequency of functional CD56^Dim^ NK cells expressing at least 2DL1, 2DL2/S2, 2DL3, 3DL1, and NKG2A and positive for all possible combinations of IFN‐γ, CCL4 and CD107a activity differed according to the functional subsets examined. The main trend that emerged from these analyses was the lower frequency of functional 2DL2/S2^+^ NK cells. When we extended these analyses to 3 SPiKIR^+^ NK cell populations using an exclusive gating strategy we found that a higher frequency of educated, compared to uneducated 3DL1^+^ and 2DL1^+^ NK cells, responded to ADNKA by secreting IFN‐γ and CCL4, but not by degranulation. In contrast, the frequency of educated, compared to uneducated, 2DL3^+^ NK cells stimulated by ADNKA did not differ significantly, with the exception of those expressing CD107a, where uneducated 2DL3 NK cells were more responsive than their educated counterparts.

We showed previously that the %GzB^+^ CEM cells generated by effector cells did not differ based on whether these cells came from individuals carrying, or not, educating *KIR‐HLA* combination for 3DL1^42^ and confirmed here that this was also the case when results were stratified for iKIR‐HLA 2DL1 and 2DL2/2DL3 pairs. We verified that the ADCC‐GTL effector cell within PBMCs were indeed NK cells that would have been subject to the effects of education.^52^ However, NK cells in PBMC effector populations have a complex effector cell make up that could mask the effect of NK cells expressing any one iNKR. This is what prompted us to isolate NK cell populations expressing single iNKR to more effectively ascertain the effect of NK cell education on ADCC‐GTL activity. CEM cells, which have the following HLA type: A*01:01, A*31:01, B*08:01, B*40:01, C*03:04, and C*07:01, were used as target cells in these ADCC‐GTL assays. Thus, CEM cells have no ligands for 3DL1 or 2DL1 but do have ligands for 2DL3.^42^, ^53^ If education were playing a role in ADCC activity it would be expected that educated self‐SP2DL1^+^ NK cells would perceive CEM cells expressing no C2 ligands as missing self and respond to these targets more robustly than did uneducated nonself‐SP2DL1^+^ NK cells. Because both educated and uneducated SP2DL1^+^ cells generated frequencies of GzB^+^ CEM that did not differ significantly from each other, this was not observed. There was also no difference in the ability of self‐ and nonself‐SP2DL3^+^ NK cells to generate GzB^+^ CEM cells. This was despite CEM cells expressing C1 alleles that can engage educated self‐SP2DL3^+^ NK cells to inhibit their activity more effectively than they could inhibit the activity of nonself‐SP3DL1^+^ NK cells. As CEM cells express no Bw4 alleles, they have the potential to be seen as missing self by self‐SP3DL1^+^ NK cells, which should result in higher activation of self‐ than nonself‐SP3DL1^+^ NK cells to generate GzB^+^ CEM cells, a phenomenon that was also not observed. In sum, these analyses did not detect an effect of education on the ADCC‐GTL read out.

A possible explanation for this may be the disparate distribution of E:Ts used in the educated versus uneducated groups. Results that call this possibility into question include the finding that the %GzB^+^ CEM cells generated by E:Ts ranging from 10:1 to 1.25:1 was stable when effector cells that were >90% CD56^Dim^ NK cells were used as effector cells. Furthermore, E:T values did not correlate with the magnitude of the %GzB^+^ CEM cells generated in ADCC‐GTL assays. The lack of a difference in the %GzB^+^ CEM cells generated when self‐SPiKIR versus SPNKG2A^+^ or total NK cells at the same E:T were used as effector cells and the lack of differences in this parameter when nonself‐SPiKIR effector cells were compared to matched SPNKG2A and total NK cells further support the notion that delivery of GzB to target cells is similar over the range of E:Ts being used in these experiments. If this is the case, then comparisons between educated and uneducated SPiKIR for their ability to support ADCC activity is valid, despite there being between‐group differences in the distribution of E:Ts.

Comparisons of within individual self‐SPiKIR^+^ with their SPNKG2A^+^ effector cells at the same E:Ts revealed no between group differences. The number of educated cells in these 2 groups would be expected to be similar. NKG2A^+^ cells are educated through interactions with HLA‐E, both of which exhibit limited variation and are expressed in most individuals.^26^, ^27^ It is important to note that education through NKG2A may differ from one person to another based on whether the HLA haplotype of the subject favors delivery of MHC‐I signal sequence nonamer epitopes to ligands for NKG2A versus iKIR.^29^ We did not detect a difference in the %GzB^+^ CEM cells generated in our ADCC‐GTL experimental set up by SPNKG2A^+^ NK cells based on the T/M amino acid dimorphism at position ‐21 of the HLA signal sequence of the HLA‐B alleles expressed by the donors whose cells were used in these experiments (not shown). However, the study population used for these analyses was small, which may have precluded detecting differences based on HLA genotype.

There is a report that educated NK cells are superior to uneducated NK cells in their ability to mediate ADCC.^54^ To show this, Kristensen et al. used PBMC effector cells depleted of NKG2A and the iKIRs that would have been educated in each of the subjects studied. They found a significant reduction in ADCC activity when PBMCs depleted of educated cells were used as effector cells compared to undepleted PBMCs or educated NK cells sorted from PBMCs. Because NKRs are expressed stochastically on NK cells, this approach would have depleted all NK cells except for those that were SP for an iKIR^+^ population that was uneducated. This would represent <10% of total NK cells. It may be that the low ADCC activity of PBMCs depleted of educated NK cells was due to the depletion of most of the NK cells rather than the inability of uneducated NK cells to mediate ADCC.

In contrast to previous findings, we observed that SPiKIR expressed high frequencies of CD16 at levels similar to those seen in SPNKG2A^+^ NK cells (Table S2). Self‐ and NS‐SPiKIR stained for intracellular GzB and perforin expressed these components of the cytotoxic granule at similar intensities. This would suggest that all of these NK cell subsets would have the capacity to become activated as a result of CD16 engagement with the Fc portion of Abs bridging NK cells and CEM target cells and, once activated, to deliver similar amounts of these components to target cells. These findings are consistent with our finding that self‐ and nonself‐SP iKIR^+^ NK cells do not differ significantly in their ability to generate GzB^+^ CEM cells. The positive correlation between the %GzB^+^ CEM cells generated by the ADCC‐GTL assay with the loss gp120‐coated CEM in an assay that measure the disappearance of these target cells due to NK cell mediated ADCC supports the notion that delivery of GzB to target cells is an early step in the pathway leading to target cell death.^48^

The low frequency of CD56^Bright^NKG2A^+^ NK cells responding to ADNKA may be partly due to the median (IQR) frequency of CD16^+^CD56^Bright^ NK cells (45.3 [31.8, 55.7]%) being lower than that of CD16^+^CD56^Dim^ NK cells (85.3 [73.6, 93.4]%). This could limit the activation of CD56^Bright^ NK cells through engagement of CD16.^49^, ^55^ As 92.4 (83.9, 95)% of CD56^Bright^ NK cells also express NKG2A, an inhibitory receptor for HLA‐E, the interaction of this receptor ligand pair may transmit inhibitory signals to CD56^Bright^ NK cells that suppress their ability to respond to anti‐HIV Ab opsonized gp120 coated CEM cells.^26^, ^55^ On the other hand, the higher frequency of CD56^Dim^NKG2A^+^, compared to CD56^Bright^NKG2A^+^ NK cells, responding to ADNKA may reflect the higher frequency of CD16^+^ cells in this NK cell population that shifts the balance of inhibitory and activating signals received from the engagement of these receptors towards activation.^49^, ^55^

The CD56^Dim^2DL2/S2^+^ population was also poorly stimulated by ADNKA. It would include NK cells expressing the iKIR 2DL2 and/or the activating 2DS2 KIR receptor. These 2 KIRs, are encoded by genes in linkage disequilibrium and are usually co‐carried.^35^ The lower frequency of NK cells expressing 2DL2/S2 that respond to anti‐HIV ADNKA may be due to the contribution of the activating 2DS2^+^ NK cells to this population. The ligands for 2DS2 are unlikely to be C1 group allotypes and may bind to HLA‐A*11.^34^, ^56^, ^57^ Thus, 2DS2^+^ NK cells are probably not educated by the same ligands as 2DL2^+^ NK cells. Even if they were educated through the same ligand, their activity would be expected to be tuned down.^58^ Another factor that may contribute to the low frequency of 2DL2/S2^+^ NK cell responses to ADNKA may be that CEM cells express C1 ligands able to interact with 2DL2 to transmit inhibitory signals.^32^

The advantage of NK cells educated through 3DL1 and 2DL1 compared to their uneducated counterparts in responding to ADNKA has been reported by others.^59^, ^60^, ^61^, ^62^ However, the previously reported observations were made using an inclusive gating strategy that would not have excluded NK cells co‐expressing NKG2A or other nonstained for iKIR on 3DL1^+^ and 2DL1^+^ NK cells. The exclusive gating strategy used here detects a more restricted population of NK cells, in which the influence of NKG2A, and several other iKIR on responsiveness to HIVIG Ab opsonized gp120‐coated CEM cells is minimized. Differences between educated and uneducated, exclusively gated NK cells did not achieve statistical significance for all functional subsets. This is likely due to the small size of the study population and the lower frequencies of functional cells observed for CD107a function compared to IFN‐γ and CCL4 secretion, which in turn impacts on the comparisons made for total function.

Our findings raise questions regarding the role of CD16 engagement versus activation through missing‐self stimulation in what we have termed ADNKA. The HLA type of CEM cells should contribute to SP2DL1 and SP3DL1 activation by missing‐self mechanisms that involve the interruption of negative signaling through these iKIR. It is intriguing that educated SP2DL3 NK cells were not more responsive to ADNKA by CEM cells than their uneducated counterparts. C1^+^ CEM cells have the potential to interact with 2DL3 on educated NK cells to transmit inhibitory signals.^32^ This may account for the absence of differences in the frequency of cells responding to ADNKA characterized by IFN‐γ and CCL4 secretion and the lower frequency of educated than uneducated NK cells expressing CD107a. Other factors that may contribute to absence of differences in ADNKA for several of the educated versus uneducated SP2DL3 functional subsets include the few observations for carriers of the C2/C2 genotype. This HLA‐C group genotype is less frequent than the C1/C1 or C1/C2 genotypes.^35^ Furthermore, interactions between 2DL3 and C1 are weaker and support more modest levels of education than do 2DL2‐C1 or 2DL1‐C2 combinations.^33^, ^35^, ^63^ Overall, these results support the interpretation that NK cell education is playing a role in ADNKA by HIVIG opsonized gp120 coated CEM and that the pattern of activation of educated versus uneducated SP2DL1, SP3DL1 and SP2DL3, NK cells is consistent with missing‐self recognition of CEM cells for the first 2 populations and inhibition through recognition of self‐ligands for the 2DL3^+^ NK cells.

It is notable that the frequency of NK cells responding to opsonized gp120 coated CEM by CD107a expression did not differ based on education through 3DL1 or 2DL1. This is consistent with the absence of the effect of education through these receptors on the %GzB^+^ cells generated in the ADCC‐GTL assay.^42^

The activation of SP3DL1 and SP2DL1 by ADNKA also requires signals through CD16 because the frequency of these cells stimulated by unopsonized CEM is lower than for opsonized gp120 coated CEM. The contribution of these signals to NK cell responsiveness would be expected to be similar for educated versus uneducated SPiKIR^+^ NK cells as the frequency of CD16^+^ NK cells among these 2 groups of SP2DL1^+^, SP2DL3^+^ and SP3DL1^+^ NK cells is not significantly different.^49^

In summary, factors important in determining ADCC and ADNKA activity differ from each other. The effect of NK cell education in ADCC activity is limited. One explanation for this may be that once NK cells are activated, either by missing self or by Ab‐dependent mechanisms they can form immune synapses, kill, disengage, and go on to kill other targets.^64^ One NK cell has been estimated to kill a mean of 4 and up to 16 targets.^64^, ^65^, ^66^, ^67^ Given that most CD56^Dim^ NK cells are CD16^+^, activation through CD16 would activate a higher frequency of NK cells than activation through missing‐self recognition. Once activated, the lesser effect arising from educated NK cells may be overwhelmed by the greater frequency of NK cells activated through CD16. On the other hand, both CD16 engagement and missing‐self recognition contribute to ADNKA. By virtue of the fact that individual NK cells activated through these mechanisms can be visualized by flow cytometry it is possible to observe the effect of NK cell education in the effector cells. The consequences of these findings for HIV vaccines is that NK cell education should contribute minimally to interindividual differences in target cell lysis by ADCC. NK cells activation by Ab‐dependent HIV‐infected cell stimuli will vary depending on how NK cells are educated, the nature of the stimulatory cell and effect of HIV infection on cell surface MHC‐I expression.

## DISCLOSURE

The authors declare no conflict of interest.

## Supporting information

Supporting information

Table S1. Study subjects classified by carriage of KIR2DL1‐C2 and KIR2DL2/L3‐C1 educating pairs.

Table S2. Study subjects NK cell subset information.
