# Supplementary material for: Differential contribution of education through KIR2DL1, KIR2DL3, and KIR3DL1 to antibody‐dependent (AD) NK cell activation and ADCC
Source: J Leukoc Biol. 2019 Jan 30;105(3):551–63. doi: 10.1002/JLB.4A0617-242RRR (PMC6916277; doi:10.1002/JLB.4A0617-242RRR)
Supplement: Supplementary file 1 — Supporting information [file JLB-105-551-s001.pptx]

## Slide 1
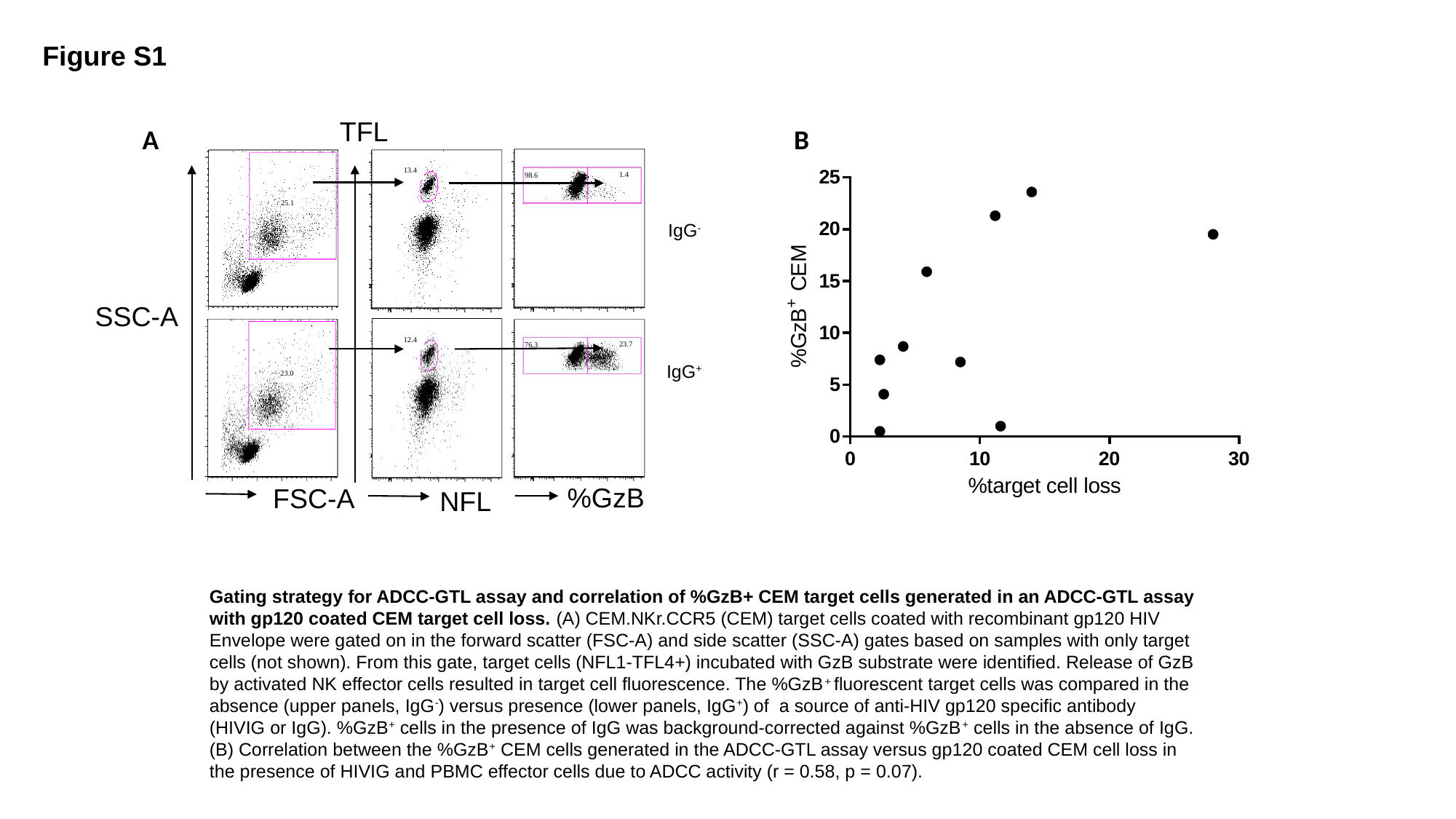

Figure S1
TFL
13.4
1.4
98.6
25.1
IgG-
SSC-A
12.4
23.7
76.3
IgG+
23.0
%GzB
FSC-A
NFL
A
B
Gating strategy for ADCC-GTL assay and correlation of %GzB+ CEM target cells generated in an ADCC-GTL assay with gp120 coated CEM target cell loss. (A) CEM.NKr.CCR5 (CEM) target cells coated with recombinant gp120 HIV Envelope were gated on in the forward scatter (FSC-A) and side scatter (SSC-A) gates based on samples with only target cells (not shown). From this gate, target cells (NFL1-TFL4+) incubated with GzB substrate were identified. Release of GzB by activated NK effector cells resulted in target cell fluorescence. The %GzB+ fluorescent target cells was compared in the absence (upper panels, IgG-) versus presence (lower panels, IgG+) of a source of anti-HIV gp120 specific antibody (HIVIG or IgG). %GzB+ cells in the presence of IgG was background-corrected against %GzB+ cells in the absence of IgG. (B) Correlation between the %GzB+ CEM cells generated in the ADCC-GTL assay versus gp120 coated CEM cell loss in the presence of HIVIG and PBMC effector cells due to ADCC activity (r = 0.58, p = 0.07).

## Slide 2
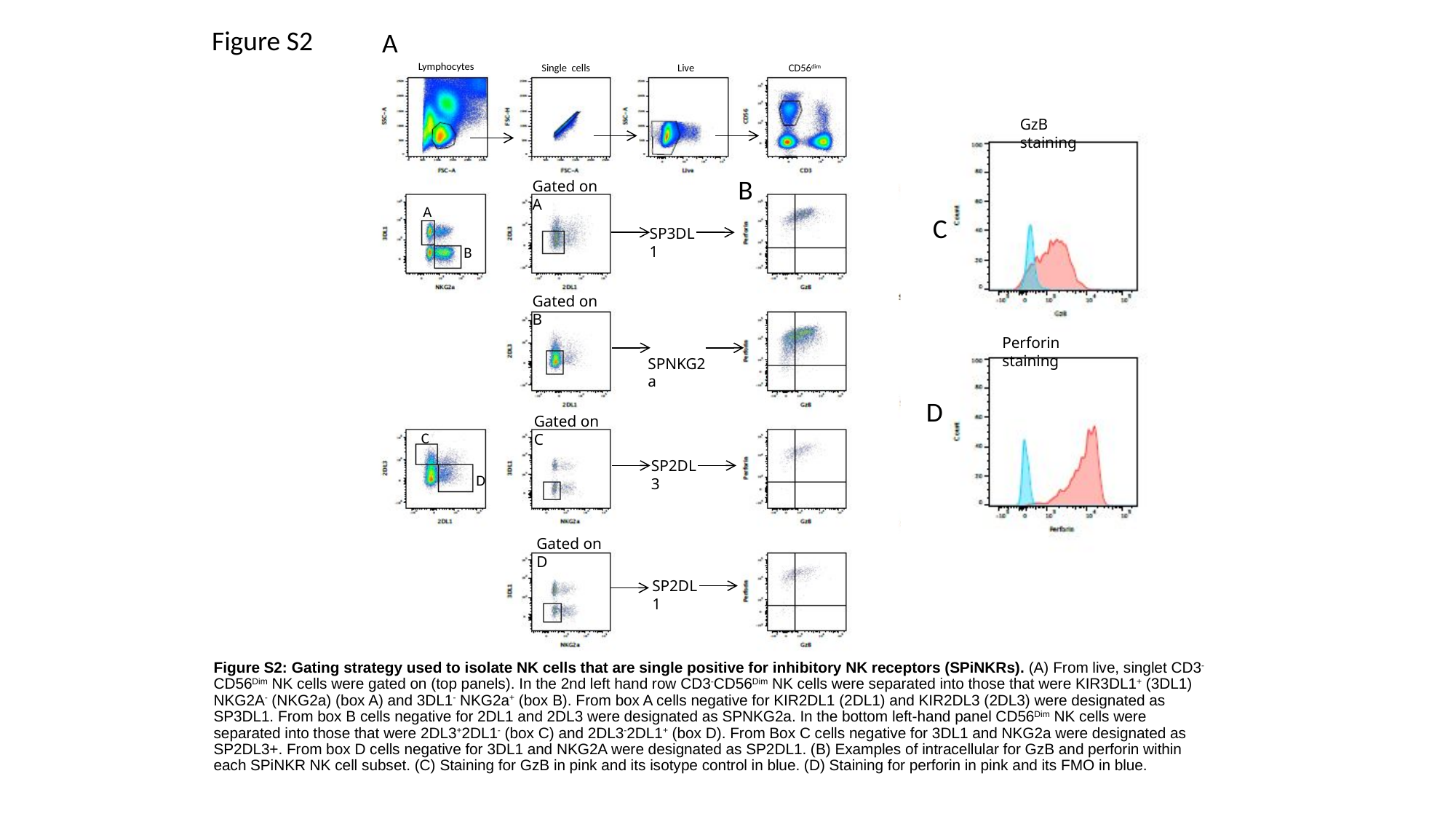

Figure S2
A
Lymphocytes
Live
CD56dim
Single cells
SP3DL1
 SPNKG2a
SP2DL3
SP2DL1
GzB staining
B) % GzB+Perforin+
Gated on CD56dim
Gated on A
B
A
Gated on A
A
C
B
B
Gated on B
Gated on B
Perforin staining
Gated on C
D
C
Gated on C
C
D
D
Gated on D
Gated on D
Figure S2: Gating strategy used to isolate NK cells that are single positive for inhibitory NK receptors (SPiNKRs). (A) From live, singlet CD3-CD56Dim NK cells were gated on (top panels). In the 2nd left hand row CD3‑CD56Dim NK cells were separated into those that were KIR3DL1+ (3DL1) NKG2A‑ (NKG2a) (box A) and 3DL1- NKG2a+ (box B). From box A cells negative for KIR2DL1 (2DL1) and KIR2DL3 (2DL3) were designated as SP3DL1. From box B cells negative for 2DL1 and 2DL3 were designated as SPNKG2a. In the bottom left-hand panel CD56Dim NK cells were separated into those that were 2DL3+2DL1- (box C) and 2DL3-2DL1+ (box D). From Box C cells negative for 3DL1 and NKG2a were designated as SP2DL3+. From box D cells negative for 3DL1 and NKG2A were designated as SP2DL1. (B) Examples of intracellular for GzB and perforin within each SPiNKR NK cell subset. (C) Staining for GzB in pink and its isotype control in blue. (D) Staining for perforin in pink and its FMO in blue.

## Slide 3
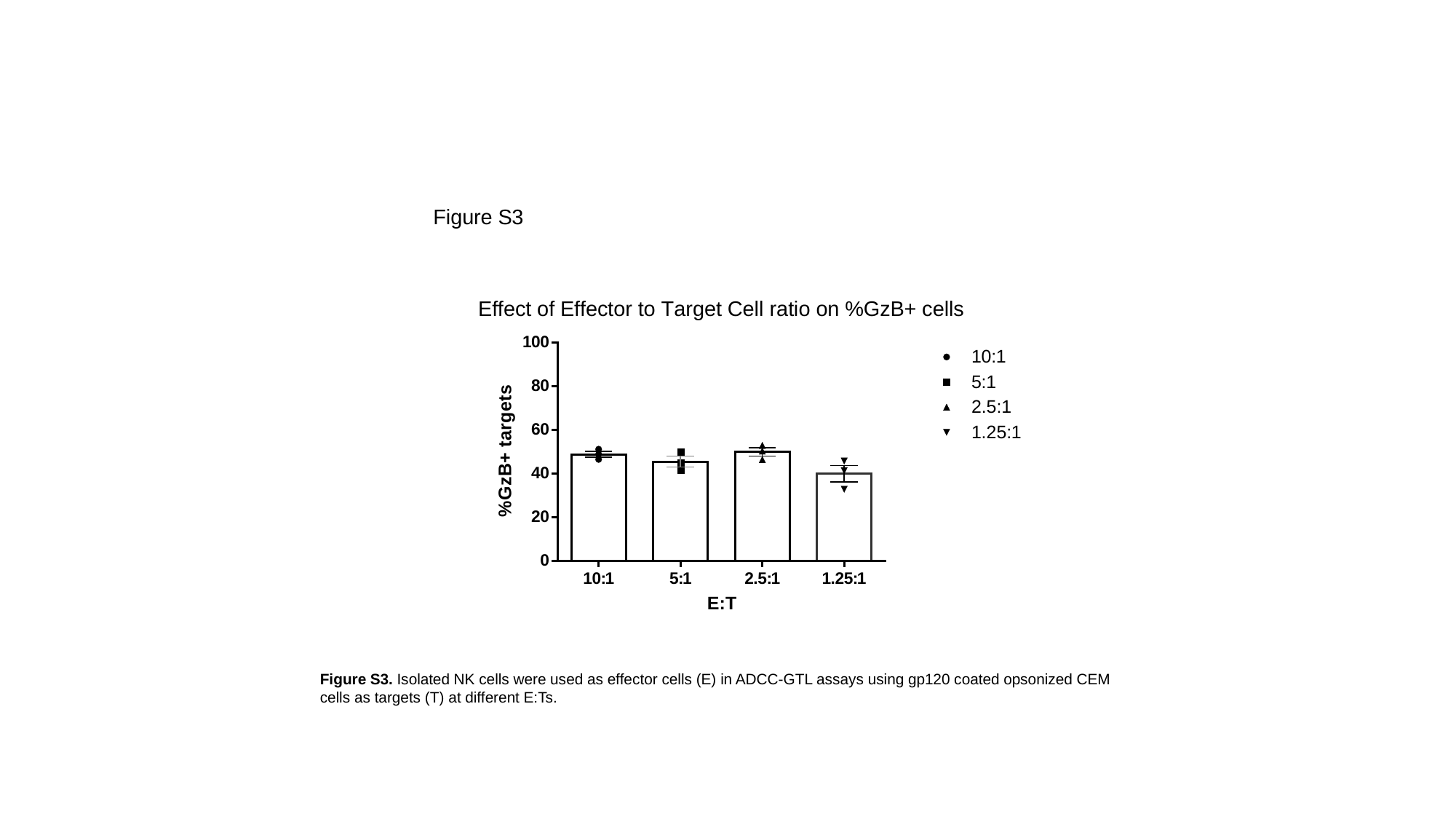

Figure S3. Isolated NK cells were used as effector cells (E) in ADCC-GTL assays using gp120 coated opsonized CEM cells as targets (T) at different E:Ts.

## Slide 4
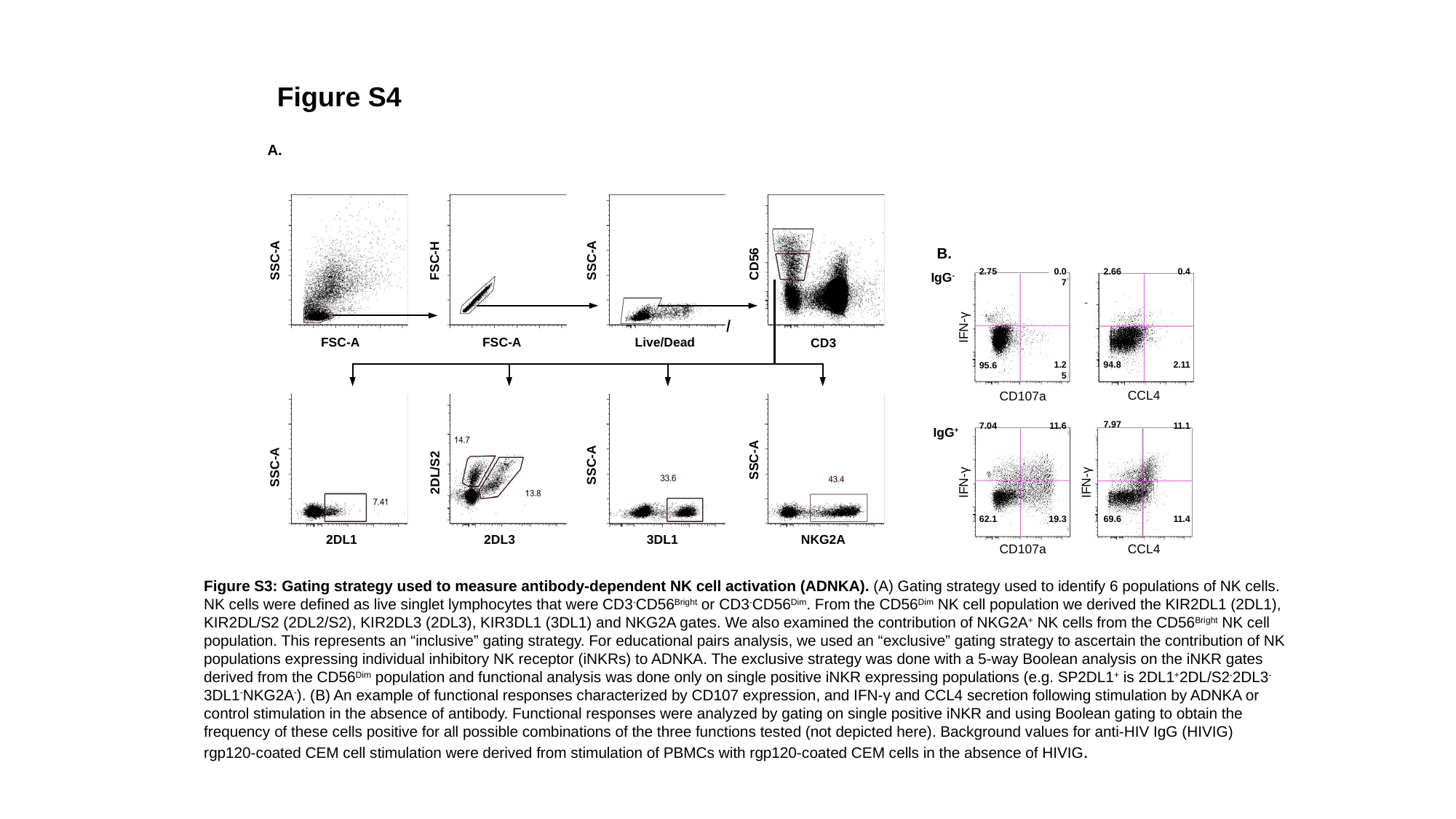

Figure S4
A.
SSC-A
FSC-A
FSC-H
FSC-A
SSC-A
Live/Dead
CD56
CD3
SSC-A
2DL1
2DL/S2
2DL3
SSC-A
3DL1
SSC-A
NKG2A
B.
IgG-
IFN-γ
CCL4
CD107a
IgG+
IFN-γ
IFN-γ
CD107a
CCL4
2.75
0.07
2.66
0.4
IFN-γ
1.25
94.8
2.11
95.6
7.04
11.6
7.97
11.1
62.1
19.3
69.6
11.4
Figure S3: Gating strategy used to measure antibody-dependent NK cell activation (ADNKA). (A) Gating strategy used to identify 6 populations of NK cells. NK cells were defined as live singlet lymphocytes that were CD3-CD56Bright or CD3-CD56Dim. From the CD56Dim NK cell population we derived the KIR2DL1 (2DL1), KIR2DL/S2 (2DL2/S2), KIR2DL3 (2DL3), KIR3DL1 (3DL1) and NKG2A gates. We also examined the contribution of NKG2A+ NK cells from the CD56Bright NK cell population. This represents an “inclusive” gating strategy. For educational pairs analysis, we used an “exclusive” gating strategy to ascertain the contribution of NK populations expressing individual inhibitory NK receptor (iNKRs) to ADNKA. The exclusive strategy was done with a 5-way Boolean analysis on the iNKR gates derived from the CD56Dim population and functional analysis was done only on single positive iNKR expressing populations (e.g. SP2DL1+ is 2DL1+2DL/S2-2DL3-3DL1-NKG2A-). (B) An example of functional responses characterized by CD107 expression, and IFN-γ and CCL4 secretion following stimulation by ADNKA or control stimulation in the absence of antibody. Functional responses were analyzed by gating on single positive iNKR and using Boolean gating to obtain the frequency of these cells positive for all possible combinations of the three functions tested (not depicted here). Background values for anti-HIV IgG (HIVIG) rgp120-coated CEM cell stimulation were derived from stimulation of PBMCs with rgp120-coated CEM cells in the absence of HIVIG.
