# Supplementary material for: Differential contribution of education through KIR2DL1, KIR2DL3, and KIR3DL1 to antibody‐dependent (AD) NK cell activation and ADCC
Source: J Leukoc Biol. 2019 Jan 30;105(3):551–63. doi: 10.1002/JLB.4A0617-242RRR (PMC6916277; doi:10.1002/JLB.4A0617-242RRR)
Supplement: Supplementary file 2 — Table S1. Study subjects classified by carriage of KIR2DL1‐C2 and KIR2DL2/L3‐C1 educating pairs. [file JLB-105-551-s002.docx]

**Table S1. Study subjects classified by carriage of KIR2DL1-C2 and KIR2DL2/L3-C1 educating pairs**.

| **Subject** | **HLA-C1/C2** | **2DL1** | **2DL1-C2** | **2DL2/2DL3** | **2DL2/L3-C1** |
| --- | --- | --- | --- | --- | --- |
| 1*^a^* | C1*^b^* | 1*^c^* | 0 | 0/1 | 1 |
| 2 | HTZ | 1 | 1 | 1/1 | 1 |
| 3 | HTZ | 1 | 1 | 0/1 | 1 |
| 4 | HTZ | 1 | 1 | 1/1 | 1 |
| 5 | HTZ | 1 | 1 | 0/1 | 1 |
| 6 | HTZ | 1 | 1 | 0/1 | 1 |
| 7 | C1 | 1 | 0 | 0/1 | 1 |
| 8 | C1 | 1 | 0 | 1/1 | 1 |
| 9 | C2 | 1 | 1 | 1/1 | 0 |
| 10 | C1 | 1 | 0 | 0/1 | 1 |
| 11 | HTZ | 1 | 1 | 0/1 | 1 |
| 12 | C1 | 1 | 0 | 1/1 | 1 |
| 13 | C2 | 1 | 1 | 0/1 | 0 |
| 14 | HTZ | 1 | 1 | 1/1 | 1 |
| 16 | HTZ | 1 | 1 | 1/1 | 1 |
| 17 | C1 | 1 | 0 | 0/1 | 1 |
| 18 | C1 | 1 | 0 | 1/1 | 1 |
| 19 | C1 | 1 | 0 | 1/1 | 1 |
| 20 | HTZ | 1 | 1 | 1/0 | 1 |
| 21 | C2 | 1 | 1 | 0/1 | 0 |
| 22 | C2 | 1 | 1 | 0/1 | 0 |
| 23 | C1 | 1 | 0 | 0/1 | 1 |
| 24 | C1 | 1 | 0 | 0/1 | 1 |
| 25 | HTZ | 1 | 1 | 0/1 | 1 |
| 26 | HTZ | 1 | 1 | 0/1 | 1 |
| 27 | C1 | 1 | 0 | 1/1 | 1 |
| 28 | HTZ | 1 | 1 | 0/1 | 1 |
| 29 | C1 | 1 | 0 | 1/1 | 1 |
| 30 | HTZ | 1 | 1 | 0/1 | 1 |
| 31 | C1 | 1 | 0 | 1/1 | 1 |
| 32 | HTZ | 1 | 1 | 0/1 | 1 |
| 33 | C1 | 1 | 0 | 1/1 | 1 |
| 34 | C1 | 1 | 0 | 1/1 | 1 |
| 35 | HTZ | 1 | 1 | 1/1 | 1 |
| 37 | C1 | 1 | 0 | 1/0 | 1 |
| 38 | HTZ | 1 | 1 | 1/1 | 1 |
| 39 | C1 | 1 | 0 | 0/1 | 1 |
| 40 | HTZ | 1 | 1 | 1/1 | 1 |
| 41 | HTZ | 1 | 1 | 1/1 | 1 |
| 43 | C2 | 1 | 1 | 1/1 | 0 |
| 44 | C2 | 1 | 1 | 1/1 | 0 |
| 45 | HTZ | 1 | 1 | 0/1 | 1 |
| 46 | C2 | 1 | 1 | 0/1 | 0 |
| 47 | HTZ | 1 | 1 | 0/1 | 1 |
| 48 | HTZ | 1 | 1 | 1/1 | 1 |
| 49 | HTZ | 1 | 1 | 0/1 | 1 |
| 50 | C2 | 1 | 1 | 1/1 | 0 |
| 51*^d^* | C2 | 1 | 1 | 1/1 | 0 |
| 52 *^d^* | C2 | 1 | 1 | 0/1 | 0 |
| 53 *^d^* | C1 | 1 | 0 | 0/1 | 1 |
| 54 *^d^* | C1 | 1 | 0 | 0/1 | 1 |

*^a^* Codes for subjects #1 through 50 are the same as those used in Table 1 of reference 26, which provides information on HLA-A, -B and -C typing. PBMCs from these subjects were used as effector cells in antibody dependent cellular cytotoxicity-GranToxiLux (ADCC-GTL) assays.

*^b^* C1=HLA-C1 group homozygous; C2= HLA-C2 group homozygous; HTZ = HLA-C1/C2 group heterozygous.

*^c^* 0=absent; 1=present.

*^d^* PBMC from subjects 51 to 54 were not used as PBMC effector cells in ADCC-GTL assays. These subjects contributed PBMCs used in either antibody dependent NK cell activation assays (#51) or for the isolation of single positive inhibitory NK receptor positive NK cells for use as effector cells for ADCC-GTL assays (#52-54).
