# Supplementary material for: Differential contribution of education through KIR2DL1, KIR2DL3, and KIR3DL1 to antibody‐dependent (AD) NK cell activation and ADCC
Source: J Leukoc Biol. 2019 Jan 30;105(3):551–63. doi: 10.1002/JLB.4A0617-242RRR (PMC6916277; doi:10.1002/JLB.4A0617-242RRR)
Supplement: Supplementary file 3 — Table S2. Study subjects NK cell subset information. [file JLB-105-551-s003.docx]

**Table S2. Study subjects NK cell subset information**.

| **Subject** | **%NK cells*^a^*** | **%GzB+**  **CEM** | **ADNKA*^b^*** | | **%CD16*^c^*** | **%NKG2A*^c^*** | **%SP2DL1*^d^*** | | **%CD16+**  **SP2DL1*^e^*** | **%SP2DL3*^d^*** | **%CD16+**  **SP2DL1 *^e^*** | **%SP3DL1 *^d^*** | **%CD16+**  **SP3DL1 *^e^*** |
| --- | --- | --- | --- | --- | --- | --- | --- | --- | --- | --- | --- | --- | --- |
| 1*^f^* | 4.57 | 30.5 | |  |  |  | |  |  |  |  |  |  |
| 2 | 6.84 | 23.6 | | Yes |  |  | | 4.14 |  | 8.54 |  | 3.99 |  |
| 3 | 3.77 | 21.3 | | Yes |  |  | |  |  |  |  |  |  |
| 4 | 8.18 | 20.4 | |  |  |  | |  |  |  |  |  |  |
| 5 | 40.2 | 17.4 | |  | 98.1 | 15.1 | | 3.2 | 94.3 | 6.4 | 49.5 | 9.2 | 93.2 |
| 6 | 5.67 | 15.6 | | Yes |  | 31.4 | | 4.7 | 96.9 | 4.7 | 96.9 | 7.4 | 98.8 |
| 7 |  | 15.5 | |  |  |  | |  |  |  |  |  |  |
| 8 |  | 14.2 | |  |  |  | |  |  |  |  |  |  |
| 9 | 5.35 | 10.6 | |  |  |  | |  |  |  |  |  |  |
| 10 |  | 9.8 | |  |  |  | |  |  |  |  |  |  |
| 11 | 5.16 | 8.8 | |  | 91.1 | 30.9 | |  |  |  |  |  |  |
| 12 | 2.97 | 7.4 | |  | 93.4 | 50.8 | | 1.8 | 98.1 | 7.4 | 98.4 | 8.0 | 98.7 |
| 13 |  | 6.7 | |  |  |  | |  |  |  |  |  |  |
| 14 | 5.12 | 6.7 | |  |  |  | |  |  |  |  |  |  |
| 16 | 2.49 | 4.0 | |  |  |  | |  |  |  |  |  |  |
| 17 | 12.0 | 1.4 | |  | 92.1 | 51.8 | |  |  |  |  |  |  |
| 18 | 4.9 | 0.5 | |  |  |  | |  |  |  |  |  |  |
| 19 | 2.41 | 0.3 | |  |  |  | |  |  |  |  |  |  |
| 20 | 2.35 | -1.2 | |  |  |  | |  |  |  |  |  |  |
| 21 | 6.87 | 12.4 | | Yes |  | 34.9 | | 13.1 |  | 4.1 |  | 3.9 |  |
| 22 | 7.1 | 10.8 | |  |  |  | |  |  |  |  |  |  |
| 23 | 1.36 | 27.3 | | Yes |  |  | |  |  |  |  |  |  |
| 24 | 9.09 | 21.4 | |  | 84.6 | 70.6 | |  |  |  |  |  |  |
| 25 | 8.87 | 21.4 | |  |  |  | |  |  |  |  |  |  |
| 26 | 7.29 | 19.5 | |  | 40.6 | 70.7 | |  |  |  |  |  |  |
| 27 | 5.5 | 18.6 | |  |  |  | |  |  |  |  |  |  |
| 28 |  | 17.9 | |  |  |  | |  |  |  |  |  |  |
| 29 | 6.15 | 16.7 | | Yes |  |  | | 4.3 |  | 1.6 |  | 19.9 |  |
| 30 | 8.08 | 15.9 | | Yes |  | 30.2 | | 7.3 | 97.3 | 4.3 | 90.3 | 5.2 | 92.7 |
| 31 | 3.03 | 12.8 | | Yes | 58.3 | 63.7 | |  |  |  |  |  |  |
| 32 | 1.14 | 7.2 | | Yes |  | 26.3 | | 3.6 | 98.3 | 8.4 | 98.3 | 11.6 | 98.7 |
| 33 | 1.96 | 7.2 | |  | 85.3 | 43 | |  |  |  |  |  |  |
| 34 | 12.5 | 6.0 | | Yes |  |  | | 3.75 |  | 7.3 |  | 4.14 |  |
| 35 | 3.01 | 5.6 | |  | 96.6 | 61.8 | |  |  |  |  |  |  |
| 37 | 1.37 | 4.3 | |  | 84.1 | 48.35 | |  |  |  |  |  |  |
| 38 | 3.82 | 4.1 | | Yes |  |  | |  |  |  |  |  |  |
| 39 | 2.44 | 3.1 | |  | 58 | 62.8 | |  |  |  |  |  |  |
| 40 | 1.83 | 2.9 | |  |  |  | |  |  |  |  |  |  |
| 41 | 2.31 | 1.4 | |  |  |  | |  |  |  |  |  |  |
| 43 | 4.21 | 27.0 | |  |  |  | |  |  |  |  |  |  |
| 44 |  | 26.5 | | Yes |  |  | |  |  |  |  |  |  |
| 45 | 1.61 | 21.3 | | Yes |  |  | |  |  |  |  |  |  |
| 46 | 3.65 | 15.1 | |  |  |  | |  |  |  |  |  |  |
| 47 | 9.39 | 12.8 | |  | 73.6 | 71 | |  |  |  |  |  |  |
| 48 | 8.7 | 7.34 | | Yes |  |  | |  |  |  |  |  |  |
| 49 | 1.15 | 5.5 | | Yes |  |  | | 7.5 |  | 4.4 |  | 17 |  |
| 50 | 1.38 | -0.6 | | Yes |  |  | |  |  |  |  |  |  |
| 51 | 3.32 |  | | Yes | 94.8 | 35.6 | | 20 |  | 4.1 |  | None^7^ |  |
| 52 |  |  | |  |  | 23.7 | |  |  |  |  |  |  |
| 53 |  |  | |  |  | 25.1 | | 8.6 | 92.4 | 8.2 | 92.4 | 1.8 | 90.5 |
| 54 |  |  | |  |  | 15.1 | | 16.5 | 97.6 | 13.1 | 97.6 | None*^g^* | None |
| Median  (IQR)*^h^* |  |  | |  | 85.3  (74, 93) | \| 39.3 \| \| --- \| \| (27, 63) \| \|  \| | | 4.7  (3.7, 10.8) | 97.3  (96, 98) | 6.4  (4.2, 8.3) | 96.9  (91.3, 98) | 7.4  (4, 11.6) | 96  (93, 99) |

*^a^* Frequency of NK cells within the live singlet lymphocyte population.

*^b^* Donors used in the antibody dependent NK cell activation (ADNKA) assays.

*^c^* Frequency within the CD56^Dim^ population.

*^d^* Frequency of single positive KIR2DL1 (%SP2DL1), KIR2DL3 (%SP3DL3) and KIR3DL1 (%SP3DL1) within the CD56^Dim^ NK cell population.

*^e^* Frequency of CD6+ cells within the SP2DL1, SP3DL3 and SP3DL1 CD56^Dim^ NK cell populations.

*^f^* Subject codes are the same as those used in Table S1.

*^g^* *KIR3DS1* homozygous subject, i.e. negative for KIR3DL1.

*^h^* IQR = Interquartile range.
